# Flammability and Thermoregulation Performance of Multilayer Protective Clothing Incorporated with Phase Change Materials

**DOI:** 10.3390/ma17235826

**Published:** 2024-11-27

**Authors:** Muhammad Shoaib, Hafsa Jamshaid, Rajesh Kumar Mishra, Kashif Iqbal, Miroslav Müller, Vijay Chandan, Tatiana Alexiou Ivanova

**Affiliations:** 1 School of Engineering and Technology, National Textile University, Sheikhupura Road, Faisalabad 37610, Pakistan; ranashoaib.shoaib@gmail.com (M.S.); kashif.iqbal@ntu.edu.pk (K.I.); 2 Department of Material Science and Manufacturing Technology, Faculty of Engineering, Czech University of Life Sciences Prague, Kamycka 129, Suchdol, 165 00 Prague, Czech Republic; muller@tf.czu.cz (M.M.); vijay@tf.czu.cz (V.C.); 3 Department of Sustainable Technologies, Faculty of Tropical AgriSciences, Czech University of Life Sciences Prague, Kamycka 129, Suchdol, 165 00 Prague, Czech Republic; ivanova@ftz.czu.cz

**Keywords:** fire fighters’ clothing, phase change materials, comfort, infraded thermography, thermal protective testing

## Abstract

Firefighters need personal protection equipment and protective clothing to be safe and protected when responding to fire incidents. At present, firefighters’ suits are developed by using inherently thermal-resistant fibers but pose serious problems related to comfort. In the present research, multilayered fire-fighting fabrics were developed with different fiber blends. Multilayer fire retardant (FR) fabrics with phase change materials (PCMs) inserts were developed and compared with reference multilayer fabrics without PCM. In this context, four fabric samples were chosen to fabricate the multilayer FR fabrics. Properties of multilayer fabrics were investigated, which include physical, thermo–physiological comfort, and flame-resistant performance. The heating process of the clothing was examined using infrared (IR) thermography, differential scanning calorimetry (DSC), thermal protective testing (TPP), and steady-state (Convective and Radiant) heat resistance tests. Areal density and thickness were measured as physical parameters, and air permeability (AP), overall moisture management capacity (OMMC), and thermal conductivity were measured as thermo–physiological comfort characteristics. The inclusion of PCM improved the thermal protection as well as flame resistance significantly. Sample S1 (Nomex + PTFE + Nomex with PCM) demonstrated superior fire resistance, air permeability, and thermal protection, with a 37.3% increase in air permeability as compared to the control sample (SC) by maintaining comfort while offering high thermal resilience. The inclusion of PCM enhanced its thermal regulation, moderating heat transfer. Flame resistance tests confirmed its excellent performance, while thermo–physiological assessments highlighted a well-balanced combination of thermal conductivity and air permeability. This study will help to improve the performance of firefighter protective fabrics and provide guidelines in terms of balancing comfort and performance while designing firefighter protective clothing for different climatic conditions.

## 1. Introduction

Globally, firefighters’ safety remains a critical issue, as shown by data from the US National Fire Protection Association (NFPA), which reported 62,085 firefighter injuries in 2023. A total of 39.2% of all injuries occurred at the scene of fire incidents [1]. In this perilous profession, firefighters endure strenuous physical activity amid intense heat and flames, making flame-resistant clothing essential for safeguarding their well-being [2,3]. Firefighters’ suits (FFSs) keep them safe from flames, steam, toxic gases, high temperatures, radiation, smoke, and other hazards while also allowing perspiration to escape and making them more comfortable to wear. The degree of heat intensity, the materials used, the quantity and distribution of moisture, and the design of the garments all have a role in how effectively they operate thermally and defensively [4,5,6,7,8,9]. There are three layers in firefighter suits: (a) outer layer, (b) moisture barrier, and (c) thermal barrier. Each layer of an FFS serves a specific purpose: the outer layer shields against heat and flames, the moisture barrier ensures breathability while maintaining insulation, and the thermal barrier keeps the firefighter comfortable by regulating temperature and allowing sweat to evaporate. Together, these layers help minimize heat stress and enhance performance in demanding conditions [10,11,12,13]. However, the need for comfort and safety are intrinsically incompatible with one another in many protective clothing types, most notably those worn by firefighters [14].

Comfort in FFSs is dependent on the heat and moisture transfer capability of the fabric. Regarding this, newly created firefighter apparel was investigated for thermal comfort, moisture management, and flame testing. Such apparel consists of outer shell fabric (75% meta-aramid, 23% para-aramid, and 2% antistatic woven fabric), moisture barrier fabric (85% meta-aramid, 15% para-aramid polyurethane PU membrane laminated to fire resistant (FR) nonwoven fabric), thermal liner fabric (aramid felt quilted to aramid/viscose FR nonwoven fabric), and fire-resistant underwear fabric (78% FR viscose, 20% para-aramid, 2% antistatic single jersey knitted fabric). According to the findings, the inner layer significantly impacts comfort, and, as the number of layers increases, heat and moisture transfer decreases while flame protection improves [15]. Similarly, the woven fabrics were examined with respect to flame retardancy, anti-static, and wear comfort qualities using two kinds of yarn, including modacrylic, FR-rayon, anti-static polyester (PET), and cotton fibers, with two different weaves, ripstop and 2/1 twill. In both twill and rip weaves, the FR-rayon-blended modacrylic fabric with antistatic PET fibers provided superior flame-retardant and antistatic qualities than the cotton-blended modacrylic textiles [16,17]. The effect of aerogel on the thermal protective performance (TPP) of FFS was investigated by using three layers: outer (100% meta-aramid), shell (100% meta-aramid), and thermal barrier (100% meta-aramid) layers. The results showed that aerogel-treated gear had higher TPP than standard firefighting gear [18,19,20,21].

Additionally, studies have explored moisture barrier fabrics for fire-protective clothing, incorporating additional chemical and biological protection features with varying properties. They utilized carbon fabric with a single jersey knitted structure, biaxially stretched microporous expanded polytetrafluoroethylene (ePTFE), and hot melt breathable polyurethane adhesive materials. The results concluded that the developed ePTFE-activated carbon fabric laminate shows significant potential to fulfill moisture barrier requirements for firefighter clothing, as its char length of 4.2 cm meets the necessary safety standards for such garments [22,23]. Other studies investigated the thermal properties of multilayered fabrics, incorporating two outer shell textiles, four moisture barrier fabrics, and two thermal barrier fabrics. The outer shells consisted of meta-aramid with a 2/1 twill weave and polybenzimidazole (PBI) fabric with a ripstop weave, both laminated via hot-melt lamination. The moisture barriers included waterjet nonwoven, 1/1 plain woven fabric, and single jersey knitted fabrics, while the thermal barrier was aramid/viscose FR fabric. Thermal conductivity was significantly influenced by the combination of these layers, with single-layer and multilayer PBI outer shells demonstrating superior thermal performance [24,25,26,27]. Moreover, single-layer and multilayer fabrics, including FR aramid/cotton, viscose/wool, acrylic/cotton, cotton, and polyester/cotton blends, were evaluated. The FR aramid/cotton, FR viscose/wool, and FR acrylic/cotton blends exhibited greater shrinkage compared to the FR cotton and polyester/cotton blends. Multilayer textiles showed increased fragility post-burning. Although all samples met the required standards, the aramid/cotton blend was identified as the most suitable for firefighter protective apparel [28]. However, the challenge of balancing comfort and safety remains unresolved, with limited progress in achieving effective solutions.

In this context, phase change materials (PCMs) have attracted the attention of researchers in textiles for coating or encapsulation to make thermo-regulated smart textiles. The PCM-coated multilayer fabric is a breakthrough in FFS, combining PCMs to create a dynamic thermal barrier. By absorbing and releasing heat precisely when needed, this advanced system not only enhances intrinsic flame resistance but also regulates temperature, protecting against extreme heat and providing comfort in high-stress environments [29]. Multi-layered FFS treated by PCMs used four layers with different fiber blends: the outer shell (Aramid and Kevlar blended), moisture barrier (Aramid felt with PTFE (Polytetrafluoroethylene) film), thermal barrier (Kevlar felt), and inner layer (100% cotton fabric) coated by micro-encapsulated PCMs. The result was that the thermal protection increased when the PCM coating increased [30,31]. A mathematical model was developed to predict the thermal performance of PCM multilayer FR fabrics [32]. PCM-coated multilayer fabrics show great potential, but their full effectiveness is still limited.

There is ample scope for advancements in improving PCMs and their compatibility and assembly for optimal comfort and safety. The current research is focused on the development of novel advanced apparel assemblies incorporating PCMs to assess their effectiveness in combination with fiber blend characteristics, as well as their influence on physical, thermal, flammability, and thermo–physiological comfort properties. The performance of PCM-enhanced clothing was evaluated through comparisons with standard multilayer fabric assemblies of similar geometry obtained from the market. These studies involve a detailed analysis of heat transfer mechanisms within the textile assemblies under controlled heat exposure conditions, aiming to quantify their thermal regulation and protective efficacy. These textile assemblies represent a breakthrough in creating dynamic, thermo-regulated fabrics for enhanced firefighter comfort and safety.

## 2. Materials and Methods

### 2.1. Materials

Meta Aramid/Nomex fibers were procured from the Dupont™ industry, Modacrylic Protex fibers were procured from Kanecaron OPAN, Faisalabad, Pakistan, and polyacrylonitrile (PAN)-based carbon fibers by Zoltek, Totowa, NJ, USA. Polytetraflouroethylene (PTFE) was procured from Escort Ltd., Faisalabad, Pakistan. Coconut oil as a phase change material (PCM), NaOH, and formic acid were purchased from Sigma Aldrich, St. Louis, MO, USA. The pH modifiers and binder (Laurapret N-DPS Achroma, Pratteln, Switzerland) were sourced from the Archroma industry in Pakistan. All chemicals were of analytical grade (99.5%) and used without any further purification. All fabric layers were prepared with a weave structure the same as the control/market sample and based on the literature [33,34]. The sample IDs and their composition are mentioned in Table 1.

### 2.2. Methods

#### 2.2.1. Fabrication of Multilayer Fabrics

The sample IDs and composition are specified in Table 1. Four different compositions of three-layered fabrics were prepared. Sample S1 had Nomex in the outer layers, PTFE as the center layer of multilayer fabric, and the inner layer of Nomex coated with PCM. Similarly, Samples S2, S3, and S4 were prepared with Protex fabrics, a 70/30 blend of Nomex/Carbon fabrics, and a 70/30 blend of Nomex/Protex fabrics, respectively. Stitching of the layers was performed with Nomex thread of 40 Tex by single needle lock stitch machine, needle no. 14, and a stitch density of 35/cm.

#### 2.2.2. Immobilization of PCM on the Fabric Surface

**Figure 1:**
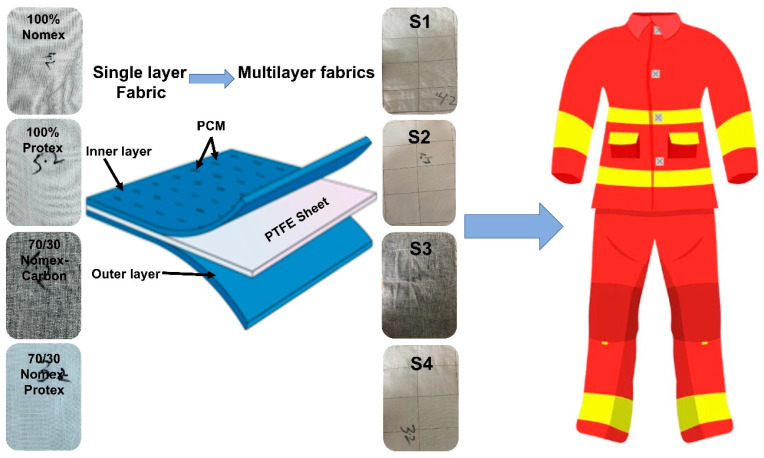
Schematic and assembly for the fabrication of multilayer firefighter suit.

Phase change materials (PCMs) are substances with the capacity to absorb and release energy in the form of latent heat, frequently using a phase transition. It will be simpler to make comfortable clothing that is also protective by using PCMs as thermo-regulating materials and fusing them into fabrics. They are good for maintaining a thermal balance by melting down while holding heat when the outside temperature rises, thereby providing comfort. The use of microencapsulated phase change materials (MPCMs) as efficient heat management devices is gaining popularity [33,34]. A PCM was applied to all the multilayer fabric samples on the top surface layer only so that any difference in performance could be compared with the other surfaces. A 500 mL volume glass beaker was filled with 200 mL distilled water and 25 g of coconut oil. Sodium Lauryl Ether Sulfate (SLS), a dispersing agent from Sigma Aldrich, USA, was added in a concentration of 0.7 g/L. After that, the beaker was heated to 70 °C in a water bath and placed under a high-speed four-blade mechanical stirrer for 45 min at 10,000 rpm speed to prepare the core emulsion. NaOH and formic acid were used as pH modifiers. These capsules were in powder form and then mixed in the polyurethane binder at a 10:1 ratio while stirring for 30 min. Then, the fabric was dipped and pad-dried at 150 °C in the Stenter frame machine. The schematic and assembly for the fabrication of a multilayer firefighter suit are shown in Figure 1.

#### 2.2.3. Characterization of the Material

Multilayer firefighters’ suit/clothing materials were investigated through physical, thermal, and thermo–physiological comfort characterizations. Thermal characteristics were observed through infrared (IR) thermography, differential scanning calorimetry (DSC), thermal protective testing (TPP), vertical and auto flame, and steady-state (Convective and Radiant) heat resistance tests. Areal density/grams per square meter (GSM) and thickness were measured as physical parameters. The air permeability (AP), overall moisture management capability (OMMC), and thermal conductivity were measured as thermo–physiological comfort characteristics.

##### Thermal Analysis

- IR Thermography

Infrared (IR) thermography is the study of thermal data collection and analysis using non-contact thermal imaging sensors. The infrared portion of the electromagnetic spectrum is used in IR thermography to locate released energy. A thermal imager converts heat (radiation from an object) into temperature and then shows an image of the temperature distribution. The name of the process is infrared thermography [35,36]. The thermal images were taken using the IR916 thermal imaging camera from Cantronic System Incorporation, Canada.

- Differential Scanning Calorimetry (DSC)

Differential Scanning Calorimetry (DSC) is a thermal analysis technique that involves calculating the rate of heat flow into or out of a sample as a function of time or temperature when the sample is exposed to a predetermined temperature range [37]. DSC analysis was performed from 10–50 °C at 2 °C/min heating rate by using DSC2500 from TA Instruments, New Castle, DL, USA. The enthalpy and phase change temperature were determined using DSC by ASTM D 3418 [38].

- Thermal Protective Performance (TPP) Device

**Figure 2:**
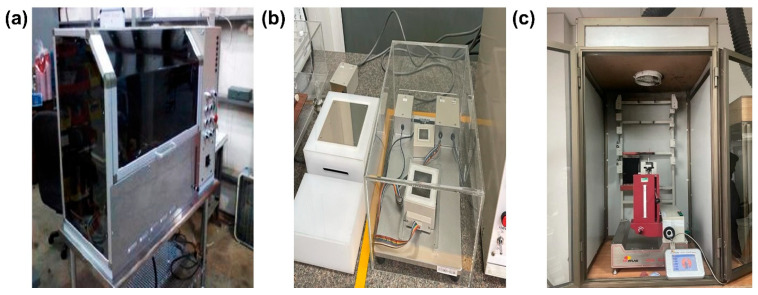
Instruments used for thermal testing of multilayer fabrics: (**a**) thermal protective tester, (**b**) Kawabata thermal conductivity tester, and (**c**) auto/horizontal flame tester.

The TPP was developed to measure how much time, convective, and radiant heat needs to travel through the multilayer fabrics before causing burns to humans. Thermal protective clothing provides defense against potentially harmful temperature conditions. Heat cannot penetrate the textile layers or the air space between them and the skin [39,40,41,42]. This test was conducted to predict the thermal protective performance of the developed fabric samples. The test was carried out according to the standard testing method ASTM-F 2700 [43]. TPP instrument is shown in Figure 2a, and the instrument utilized for the test was designed and developed by Precision Products LLC, Baoan district, Shenzhen, China.

- Vertical and Horizontal Flame Tests

A vertical flammability test was performed to analyze the burning behavior, melting, and dripping behavior of specimens. The test was performed on Vertical Flammability Tester model number M233B, from SDL Atlas, SC, USA, following the standard method ASTM-D-6413 [44]. All specimens were prepared in the standard size mentioned in the test method, and the test was performed in controlled laboratory conditions.

An auto/horizontal flame test was performed to analyze the burning behavior, hole formation, and dripping behavior of specimens. The test was performed on an auto-flame tester (SDL ATLAS), model number M233B, following the standard method ISO-6940 [45]. All specimens were prepared in the standard size mentioned in the test method, and the test was performed in controlled laboratory conditions. Five samples were tested for each variety, and the average value was calculated. The instrument of the auto flame testing is shown in Figure 2c.

- Steady-State (Convective and Radiant) Heat Resistance

The steady-state heat resistance of all individual and multilayer fabrics was evaluated, and the test was conducted to predict the thermal protective performance of the fabrics. The test was carried out according to the standard testing method ASTM D4018 [46]. The instrument utilized for the test was designed and developed at the National Textile University, Pakistan. The instrument was developed as per the standard used in the testing method. The thermocouple was used to determine the temperature gradient.

###### Physical Parameters

The physical parameters of individual and multilayer samples were evaluated before and after wet processing. The areal density/(GSM) of all fabric samples was measured using the standard method ASTM D3776 [47] by using a GSM cutter from Osaka, Japan. The thickness of samples was determined according to the standard method ASTM D1777 [48] and checked by a digital thickness machine by RMES (Rocky Mountain Engineering and Surveying, Faisalabad, Pakistan).

###### Thermo–Physiological Comfort Properties

Air permeability (AP) plays a vital role in the comfort of fabrics. The air permeability test was performed on the instrument named Air Permeability Tester SDL ATLAS model M021A following the standard method ASTM-D-737 [49].

The overall moisture management (OMMC) capability analyzes the management of the transport of liquid moisture and is investigated through a standard test method AATCC-195 [50] by using a moisture management tester MMT^®^ SDL Atlasm USA. The OMMC test comprises the measurement of wetting time, maximum wetted radius, one-way transport index, absorption rate, and spreading speed of the moisture.

The thermal conductivity of the fabrics is a very important aspect of the thermo–physiological comfort of the fabrics and was investigated by using the Kawabata evaluation system (KES-F7) shown in Figure 2b. The thermal properties of samples were tested on KES-F7 Thermo Labo II (Kato Tech. Co., Ltd., Kyoto, Japan) using the standard test method JIS L 1927:2020 [51]. Effective thermal conductivity, λ (W/mK), is calculated from the following equation:

λ = Qd/AΔT(1)

where Q is electric power to keep a steady state (Watt), d is sample thickness (m), A is the area of the heat source plate, and ΔT is the temperature difference between the heat source plate and the heat sink (Kelvin).

## 3. Results and Discussion

### 3.1. Physical Parameters of Multilayer Fabrics

The physical parameters of multilayer fabrics are given in Table S1 (Supplementary Materials). These are mean values calculated from a sample size of fifteen. The bars in Figure S1 (Supplementary Materials) show mean values with standard deviation (SD). The results are in accordance with literature [52,53].

### 3.2. Thermo–Physiological Comfort Properties of Multilayer Fabrics

The result of thermo–physiological properties is given in Table S2 (Supplementary Materials). These are mean values calculated from a sample size of ten.

Figure S2 (Supplementary Materials) shows the result of air permeability (mm/s) of all multilayer samples. The bars in Figure S2 show mean values with standard deviation (SD). Sample S1 showed 37.3% higher air permeability as compared to the control sample because the constituent Nomex fabric has a lower thickness. The control sample showed a minimum air permeability because the thickness was the highest.

The results of OMMC testing are given in Table S3 (Supplementary Materials). These are mean values calculated from a sample size of ten. The standard deviations are also given.

It can be observed very clearly that the wetting time on the top surface, which has been functionalized with PCM, shows a much lower wetting time, significantly higher absorption rate, higher wetted radius, and significantly higher spreading speed as compared to the bottom surface, which does not contain the PCMs [54,55,56,57]. Also, the OMMC parameters are far superior on the functionalized surface as compared to the control sample SC.

Figure S3 (Supplementary Materials) shows the results of the thermal conductivity (W/mk) of all multilayer samples. The bars show mean values with standard deviation (SD). The trends are supported by reported literature [58,59]. 

### 3.3. Thermal Analysis

#### Flammability Tests of Multilayer Fabrics

**Figure 3:**
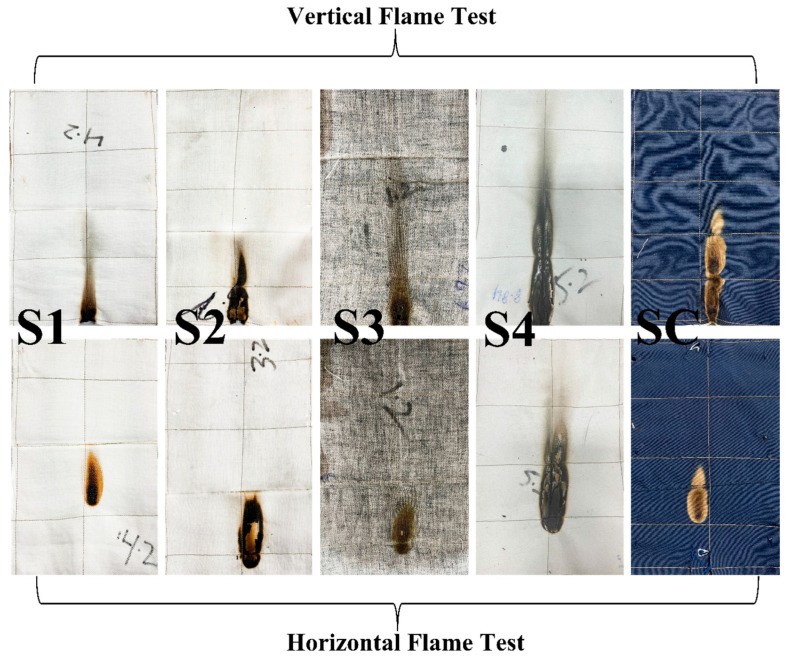
Multilayer fabrics after flame tests.

To evaluate the flame-retardant properties of multilayer fabrics, horizontal and vertical flame tests were performed, and the results are shown in Table 2 and Figure 3. These are mean values calculated from a sample size of ten. The standard deviations are also given.

After 12 s of exposure to flame in a vertical flame test, none of the samples showed any after-flame and afterglow characteristics. The char lengths of the samples tested were ordered in the following manner: S1 < S4 < S3 < SC < S2, respectively. Therefore, sample S1 showed minimum char length with high flame retardancy due to Nomex. The control sample SC showed relatively inferior results as compared to sample S1. This is because, during exposure to higher temperatures, Nomex fibers have high char ability, creating a thermal barrier that prevents burns from penetrating into the wearer’s skin [60]. Until the char hardens, this shield maintains its pliability and resilience. The convective heat transmission is reduced when the fibers expand and close the holes in the cloth. Sample S3 has the combined effect of the Nomex/Protex combined char, as Protex also has good char-forming abilities. Moreover, the remaining samples showed comparable char length to the control sample as they are also inherently flame-retardant fibers. 

Similarly, after 10 s of exposure, the horizontal flame tests were performed, and the results are summarized in Table 2 and Figure 3. All samples passed the test successfully with no hole formation. However, the control sample SC lost a higher mass as compared to the other multilayer samples developed.

### 3.4. Steady-State Heat Resistance

The result of steady-state heat resistance of fabric samples is shown in Table 3. These are mean values calculated from a sample size of ten. The standard deviations are also given. S3 is the best sample as it could resist the radiant heat for the longest time interval among all the samples, even compared to the control sample. Nomex and carbon fibers have better resistance to radiant heat [33,38]. However, in this case, it seems that fabric construction is the dominant factor. The results of convective heat resistance revealed that sample S1 showed the best results based on time intervals. The sample underwent a temperature rise in the maximum time interval. This is mainly because the aramid fibers have inherently high flame resistance [40,41]. Nomex fiber is a meta-aramid material, and it is non-crystalline in nature. Therefore, it does not conduct heat readily [42]. Carbon fibers have a very high resistance to heat due to their inherent character [43].

### 3.5. IR Thermography

**Figure 4:**
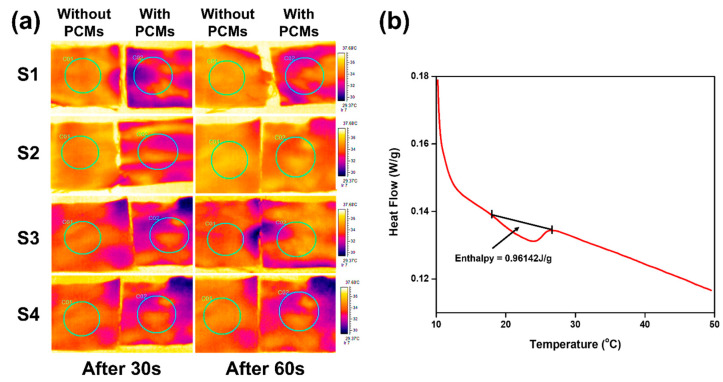
PCM’s performance: (**a**) thermal images of multilayer fabrics at different residence times. (**b**) Enthalpy of PCMs.

An infrared thermal camera has been used to study the thermal characteristics of FR fabrics. Multilayer fabric samples were prepared for thermal imaging to check the performance of the PCM that was applied on the fabric surface by the dip coat method. Both fabrics (coated and uncoated) were carefully placed on the pre-heated knob at the temperature of 40 °C. The time was recorded for both types of fabrics. The fabric samples with PCM-containing yarn showed a delay in reaching the same temperature as compared to the fabric samples without PCM [60]. The temperature rise was noted frame by frame based on time, which is shown in Figure 4a. It clearly showed that the temperature of the thermo-regulated fabric did not exceed 29 °C even after 30 s, while the fabric without PCM reached the same level in less than 10 s. The temperature of fabric with and without PCM on the spotted area exactly above the heat source has been shown after 30 and 60 s, respectively. So, it was evident that the fabric samples incorporated with PCMs provided better thermo-regulating behavior as compared to the samples without PCM.

### 3.6. Differential Scanning Calorimetry (DSC)

The enthalpy and phase change temperature of PCMs was determined using DSC by ASTM D 3418 [61], as shown in Figure 4b. A normalized enthalpy of 0.96 J/g was obtained, which specifies a good amount of energy that can be stored by the inclusion of only 1% PCM. The incorporation of PCMs in fabrics enhances its latent heat storage capacity, enabling better thermal regulation [62,63]. Figure 4b shows the melting range with a starting temperature of 18.12 °C, a peak temperature of 25.12 °C, and an ending temperature of around 28 °C.

### 3.7. Thermal Protective Performance (TPP)

**Figure 5:**
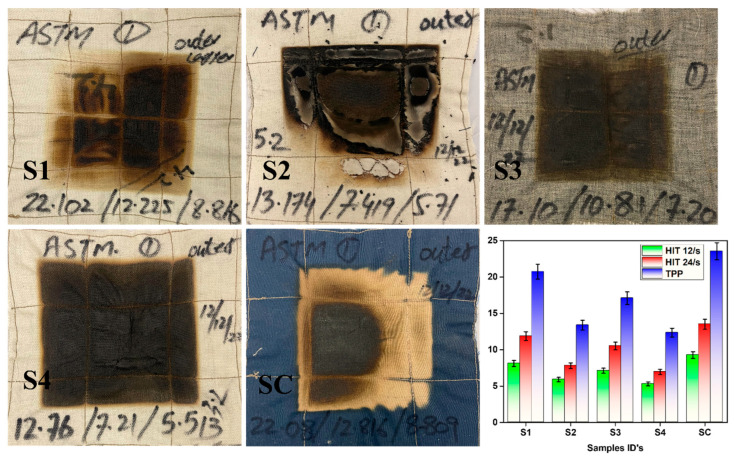
Comparison of thermal protective performance of the multilayer fabrics, S1, S2, S3, S4, SC, and barchart showing their thermal performance. The bars in Figure 5 show mean values with standard deviation (SD). As can be seen, the trend of the protective performance of the prepared samples was S1 > S3 > S2 > S4, respectively. As S1 and S3 samples consist of Nomex, which has inherently good char ability and creates a protective layer on the surface of the fabric, its thermal protective performance was better than other types of fibers used. Sample S4 showed the lowest thermal performance, which was also evident from the minimum char produced.

The objective of this measuring method is to obtain the temperature changes on the surface of human skin vs. time when exposed to the flash fire of intense heat fluxes and endeavors to obtain the correlation between the temperature rise, the amount of thermal energy exerted on the surface of human skin, and the second-degree burn injuries of the human skin tissues. The thermal protective performance of the multi-layer fabrics is shown in Table 4 and Figure 5. These are mean values calculated from a sample size of ten. The standard deviations are also given. Overall, the control sample SC showed a higher TPP value due to higher GSM and thickness as compared to the other multilayer fabrics developed. The fabric systems with high weight and thickness can trap a considerable amount of stationary or dead air within their structure. As the thermal insulation value of this stationary air is high, it does not allow easy transmission of thermal energy in the form of electromagnetic radiant-heat waves (generated from the radiant-heat source) through the fabric system [64]. This situation can enhance the thermal protective performance of the fabric system. While sample S1 has comparable results to control sample SC, with an 11.89% difference, the other samples showed lower values. This proved that Nomex fibers with PCM resulted in better thermal protective performance as compared to Protex or blends of Nomex with Protex or carbon. This can be attributed to the inherent heat resistance of the Nomex fiber material.

## 4. Conclusions

In conclusion, this study examined the physical and thermo–physiological properties of various multilayer fabric samples intended for firefighter protective clothing. The control sample form market (SC) shows the highest areal density and thickness due to the higher ends/picks density. Sample S2 (Protex + PTFE + Protex with PCM) showed the highest thermal conductivity, which was 12.5% greater than the control sample SC. This is attributed to the thermally conductive outer layer composed of Protex fibers. For air permeability, sample S1 (Nomex + PTFE + Nomex with PCM) displayed a 37.3% higher AP as compared to control sample SC, likely due to its open structure, which facilitates easier airflow. Moisture management testing revealed poor results across all samples, with a one-way transport index of zero and low spreading speeds, indicating a limited moisture-wicking capacity. Flame resistance tests showed that all samples passed the horizontal and vertical tests without afterglow or hole formation, with sample S1 (Nomex + PTFE + Nomex with PCM)) demonstrating the shortest char length in vertical testing due to the inherent char-forming properties of Nomex providing better thermal insulation in steady-state heat resistance tests. Sample S3 (70/30 Nomex/Carbon + PTFE + 70/30 Nomex/Carbon with PCM) performed the best in terms of radiant heat resistance, while sample S1 (Nomex + PTFE + Nomex with PCM) had the longest time interval in convective heat resistance, showing Nomex and carbon fibers’ resistance to heat. The control sample SC had the highest TPP value of 23.54, surpassing S1 (Nomex + PTFE + Nomex with PCM) by 11.89%, which is attributed to its high thickness and GSM, which can entrap more stationary air, enhancing thermal insulation. Balancing comfort with protection, sample S1 (Nomex + PTFE + Nomex with PCM) emerged as the top choice for firefighter clothing, effectively combining Nomex’s inherent fire resistance and sufficient air permeability to ensure comfort. With the addition of Phase Change Materials (PCMs), which help to manage heat transfer, sample S1 (Nomex + PTFE + Nomex with PCM) further enhanced its thermal regulation abilities under high-heat conditions. This study underscores that fabric assemblies crafted with Nomex, Protex, and PCM, and designed with balanced GSM and thickness, can deliver optimal thermal protection and wearing comfort. These findings emphasize the critical role of material composition and structure in advancing firefighter apparel performance. The other physical and thermal performance can be evaluated in the full clothing on manikins in the future.

## Figures and Tables

**Table 1:** Multilayer fabrics composition with their IDs.

Sample ID	Composition
S1	Nomex + PTFE + Nomex with PCM
S2	Protex + PTFE + Protex with PCM
S3	70/30 Nomex/Carbon + PTFE + 70/30 Nomex/Carbon with PCM
S4	70/30 Nomex/Protex + PTFE + 70/30 Nomex/Protex with PCM
SC	Control Sample/market sample

**Table 2:** Flame test results of the multilayer fabrics.

Horizontal Flame Test								
ID	Weight Before (g)	Weight After (g)	Weight Loss (g)	After Flame (s)	After Glow (s)	Smoke	Char Length (mm)	Bead
S1	16.02 ± 0.06	15.12 ± 0.06	0.90 ± 0.03	0	0	✔	0	✔
S2	15.53 ± 0.04	15.42 ± 0.04	0.11 ± 0.00	0	0	✔	0	✔
S3	13.92 ± 0.05	13.84 ± 0.04	0.08 ± 0.00	0	0	✔	0	✔
S4	15.41 ± 0.06	15.30 ± 0.05	0.11 ± 0.01	0	0	✔	0	✔
SC	19.95 ± 0.07	18.90 ± 0.06	1.05 ± 0.04	0	0	✔	0	✔
**Vertical Flame Test**								
**ID**	**Weight Before (g)**	**Weight After (g)**	**Weight Loss (g)**	**After Flame (s)**	**After Glow (s)**	**Smoke**	**Char Length (mm)**	**Bead**
S1	16.02 ± 0.05	15.92 ± 0.03	0.10 ± 0.00	0	0	✔	15 ± 0.5	✔
S2	15.53 ± 0.05	14.40 ± 0.02	1.13 ± 0.05	0	0	✔	30 ± 0.8	✔
S3	13.92 ± 0.04	13.77 ± 0.05	0.15 ± 0.00	0	0	✔	22 ± 0.4	✔
S4	15.41 ± 0.05	15.26 ± 0.05	0.15 ± 0.00	0	0	✔	17 ± 0.6	✔
SC	19.95 ± 0.04	19.86 ± 0.05	0.09 ± 0.00	0	0	✔	25 ± 0.7	✔

**Table 3:** Steady-state heat resistance properties of multilayer fabrics.

ID	Radiant Heat Time (min:s)	Radiant Heat Temp °C	Convective Heat Time (min:s)	Convective Heat Temp °C
S1	4:22 ± 0:05	15 ± 0.4	23:28 ± 0:03	15 ± 0.2
S2	8:10 ± 0:06	15 ± 0.4	12:19 ± 0:02	15 ± 0.4
S3	16:50 ± 0:21	15 ± 0.5	14:52 ± 0:03	15 ± 05
S4	3:01 ± 0:03	15 ± 0.3	10:33 ± 0:05	15 ± 0.4
SC	4:45 ± 0:04	15 ± 0.5	16:38 ± 0:08	15 ± 0.1

**Table 4:** Thermal protective performance analysis of the multilayer fabrics.

ID	Thickness (mm)	Sample Weight (g)	HIT12/s	HIT24/s	TPP
S1	1.16 ± 0.06	12.89 ± 0.05	8.12 ± 0.02	11.88 ± 0.06	20.74 ± 0.08
S2	1.18 ± 0.05	13.37 ± 0.04	5.91 ± 0.01	7.82 ± 0.04	13.38 ± 0.03
S3	1.12 ± 0.04	12.86 ± 0.03	7.11 ± 0.01	10.52 ± 0.06	17.10 ± 0.05
S4	1.14 ± 0.04	12.61 ± 0.02	5.31 ± 0.01	6.96 ± 0.03	12.34 ± 0.04
SC	1.19 ± 0.06	16.42 ± 0.03	9.27 ± 0.02	13.52 ± 0.07	23.54 ± 0.09

## Data Availability

The original contributions presented in this study are included in the article and Supplementary Materials, further inquiries can be directed to the corresponding author/s.

## Supplementary Materials

The following supporting information can be downloaded at: https://www.mdpi.com/article/10.3390/ma17235826/s1, Tables S1–S2 and Figures S1–S3 are given in supplementary material. References [52,53,54,55,56,57,58,59] are cited in the Supplementary Material.
