# Supplementary material for: Flammability and Thermoregulation Performance of Multilayer Protective Clothing Incorporated with Phase Change Materials
Source: Materials (Basel). 2024 Nov 27;17(23):5826. doi: 10.3390/ma17235826 (PMC11641941; doi:10.3390/ma17235826)
Supplement: Supplementary file 1 [file materials-17-05826-s001.zip › materials-3311686-supplementary.pdf]

materials

MDPI

Supplementary Materials

Table S1. Physical parameters of all multilayer samples.

Sample ID	Areal Density/GSM (g/m^2)	Thickness (mm)
S1	398±19	1.16±0.06
S2	471±21	1.18±0.05
S3	436±18	1.12±0.04
S4	481±20	1.14±0.04
SC	543±21	1.19±0.06

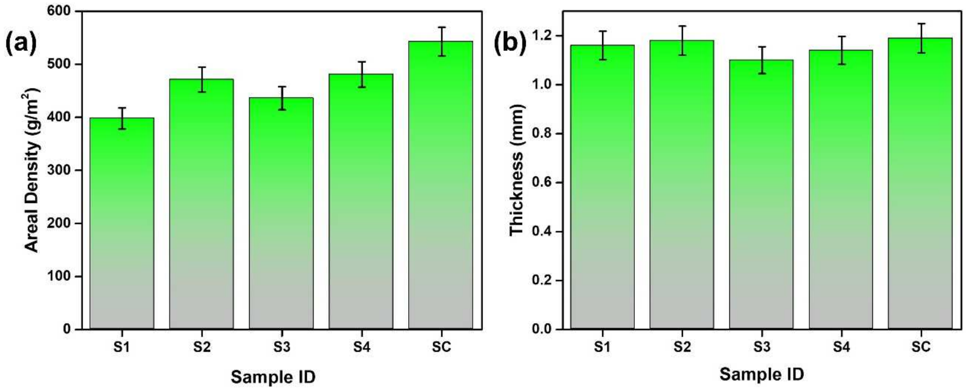

Figure S1. Physical parameters of the multilayer fabrics for firefighters (**a**) Areal density/GSM (**b**) Thickness.

3.1.1. Areal density/grams per square meter (GSM)

The areal density (g/m^2) of multilayer fabric samples is shown in Figure S1(a). The sample S4 has a maximum GSM among the developed fabrics because the outer layers were made from 70/30 Nomex/Protex fabric which has the maximum GSM. It seems that the blend of Nomex and Protex was very effective to increase the cohesivity among these two types of fibers and increasing the overall density of the fabric as compared to their other blends. The control sample from market showed a higher GSM (543±21) as compaed to all the developed fabrics because the thread density (ends/cm and picks/cm) of the control sample was inherently higher.

3.1.2. Thickness

Figure S1(b) shows the result of the thickness (mm) of all multilayer samples. Sample S2 showed maximum value of thickness because the Protex fibers are acrylic based and have lowest density among the materials used. Thus the fabrics are bulkier with a higher thickness. Thickness and GSM/areal density are closely related to each other in many cases. The results also confirmed that the samples having higher areal density resulted in higher thickness values in most cases [52]. The only exception was in sample S2 since it was composed of 100% Protex fibers in the outer layers. The control sample showed higher thickness than the developed samples because it was having inherently higher thread density (ends/cm and picks/cm). A higher thread density not only increased GSM but also reflected in higher thickness [53].

However, a direct relationship between GSM and thickness cannot be established since all the samples were prepared with different blend compositions.

3.2. Thermo-physiological Comfort Properties of Multilayer Fabrics

Table S2. Thermo-physiological characteristics of all multilayer fabric samples.

Sample ID	AP (mm/s)	OMMC	Thermal conductivity (W/mK)
S1	7.62±0.38	0	0.17±0.01
S2	6.81±0.31	0	0.18±0.01
S3	6.75±0.30	0	0.16±0.01
S4	5.84±0.29	0	0.15±0.01
SC	5.55±0.26	0	0.16±0.01

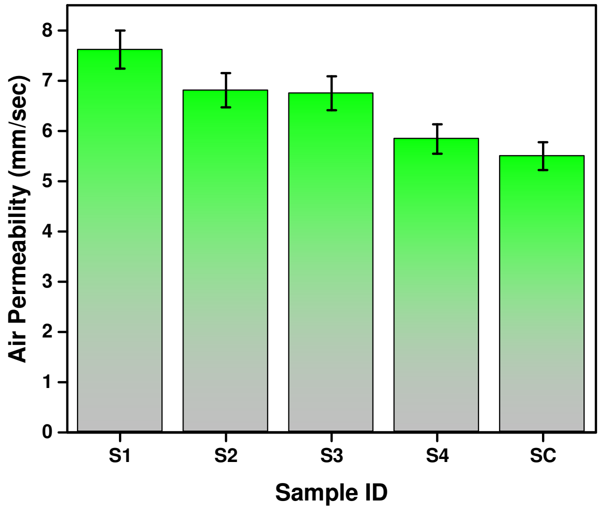

Figure S2. Air permeability of all multilayer samples.

Moisture Management of Multilayer Fabrics

A moisture management tester (MMT) was used to examine moisture management qualities. The wearer experiences the comfort of clothes influenced by the absorption of sweat from the skin through the clothing, and the movement of moisture through the microclimate across and through the fabric [54,55]. The top and bottom sides of the material begin to become wet during the few seconds-long wetting durations on top (WTt) and bottom (WTb), surfaces respectively. OMMC is measured from the slope of curve plotted between time in seconds and the total water content on the top and bottom surfaces beyond tan (15°) [56].

Table S3. Moisture Management properties of all multilayer fabric samples.

ID	Wetting Time Top (s)	Wetting Time Bottom (s)	Top Absorption Rate (%/s)	Bottom Absorption Rate (%/s)	Top Max Wetted Radius (mm)	Bottom Max Wetted Radius (mm)	Top Spreading Speed (mm/s)	Bottom Spreading Speed (mm/s)	Accumulative One-way transport index (%)	OMMC
S1	2.76±0.13	119.95±5.12	25.47±1.08	0	20.0±1.08	0	3.01±0.01	0	-716.81	0
S2	6.28±0.27	119.95±4.88	23.75±1.05	0	5.0±0.28	0	0.78±0.01	0	-523.57	0
S3	8.40±0.35	114.80±5.09	41.57±1.51	2.65	17.5±0.85	5±0.04	3.67±0.01	0.21	-683.23	0
S4	9.00±0.43	119.95±4.36	39.80±1.65	0	15.0±0.66	0	1.38±0.01	0	-529.86	0
SC	7.41±0.32	119.95±4.16	21.31±1.03	0	5.0±0.23	0	0.66±0.01	0	-411.84	0

The results are based on the moisture-management indices which classify different fabrics into standard MMT categories. The overall moisture management of the fabric provides a clue as to how it will perform in a certain circumstance [57]. Absorption of samples S1, S2 & SC fell into grade two and S3 & S4 into grade 3. The maximum wetted radius of sample S1 matched to grade 4. For samples S2, S4, and SC it matched grade 1. For sample S3, it was grade 3. Spreading speed of all samples was in grade 1. One-way transport capacity was very poor. Overall moisture management was in grade 1, which is very poor.

Thermal Conductivity of Multilayer Fabrics

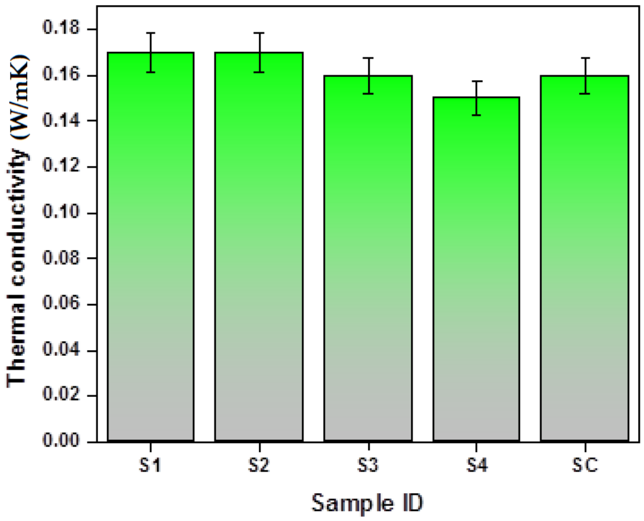

Figure S3. Thermal conductivity of all multilayer samples.

Thermal conductivity is the quantity of heat that flows through a unit area of a unit thickness in a unit time. In multi-layered fabric constructions, there are more fibers per unit area, and the thermal conductivity values are higher because the fabric is thicker [58]. Sample S2 showed 12.5% higher thermal conductivity as compared to control sample SC because the outer layer is thermally conductive. The highest thermal conductivity values were observed in the sample S2 with a 100 % Protex in the outer layers. Protex fabric sample is made from modacrylic fibers which is a specialy modified acrylic fiber, and is highly blendable with other fibers. It entraps hogher amount of air in the bulk and resulted in enhanced thermal conductivity [59]. The modacrylic fibers also have a higher heat retention characteristic. The control sample SC, showed a lower thermal conductivity value because GSM is relatively higher. The developed multilayer structures contained a higher amount of air, so-called “pockets”, which create an additional shield as a heat insulator.
